# 906. Risk of Shingles among Patients Aged 45–49 versus 50–54 Years in the US, by Immune Status

**DOI:** 10.1093/ofid/ofad500.951

**Published:** 2023-11-27

**Authors:** Amanda C Miles, Leah McGrath, Deepa Malhotra, Verna Welch

**Affiliations:** Pfizer, New York, New York; Pfizer, New York, New York; Pfizer, New York, New York; Pfizer, New York, New York

## Abstract

**Background:**

Shingles burden has historically focused on older adults, with the currently licensed vaccine in the US approved for adults aged ≥50 years. Our study aimed to estimate the incidence of shingles among adults aged 45–49 years and to ascertain whether the adjusted incidence rate (IR) was similar to adults aged 50-54 years, stratified by immune status.

**Methods:**

We conducted a retrospective analysis of de-identified Optum Clinformatics® Data Mart insurance claims to identify patients in the US diagnosed with shingles each year between 2015–2021. Adults aged 45–54 were included if they were enrolled on January 1 of a given year (index date), had at least 1 year of prior continuous enrollment, and had no shingles diagnosis in the 90 days prior. Adults could contribute to multiple calendar years, and were followed from index until the earliest of 1) shingles diagnosis, 2) disenrollment, 3) December 31 of the calendar year, or 4) death. Yearly IRs were calculated using Poisson regression models and combined time period IRs used generalized estimating equations (GEE). Models were adjusted for demographics, comorbidities, and healthcare utilization and stratified by immunocompromised (IC) status at baseline. IRs were reported as events per 100,000 person-years.

**Results:**

A total of 9.4 million unique patient-year combinations were identified in the database. Patient demographics for 2021 are shown in Table 1. The IR of shingles from 2015–2021 was approximately two-times higher for IC versus immunocompetent patients, and rates were similar for adults aged 45–49 and 50–54 (Figure 1). During the same period, IRs in immunocompetent patients aged 45–49 and 50–54 were 489.3 (95% CI: 480.6, 498.2) and 579.1 (95% CI: 569.1, 589.3) respectively. However, the difference between the IRs for the two age groups narrowed over time. The shingles IR among adults aged 45–49 in 2021 was comparable to the IRs for pneumococcal pneumonia observed when a vaccine recommendation was made for pneumonia in high-risk populations (Figure 2).

**Figure:**
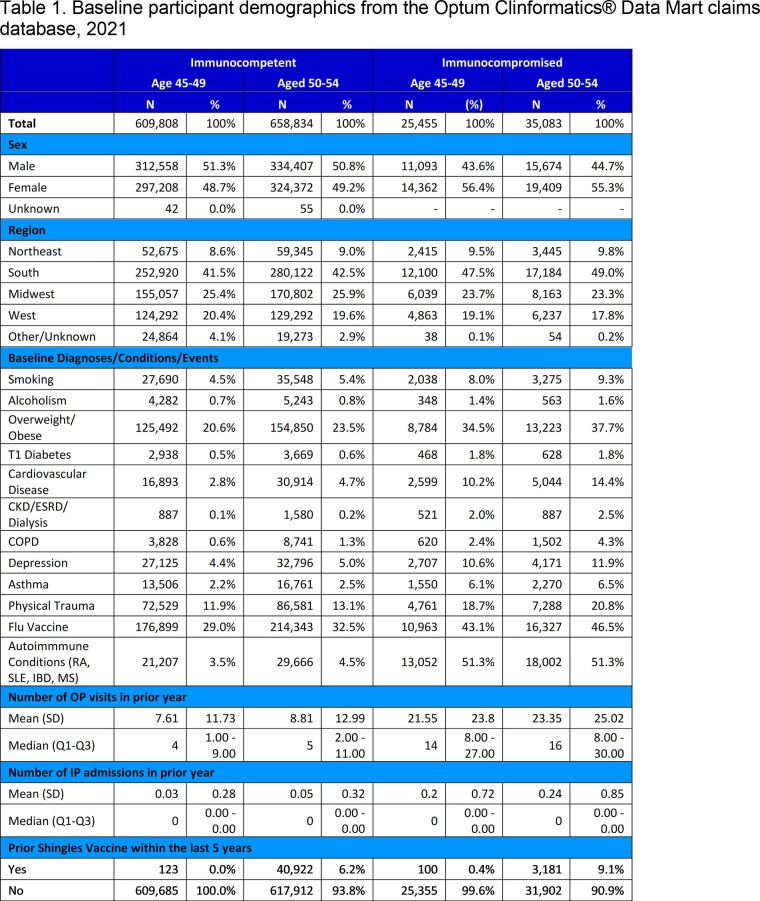

**Figure:**
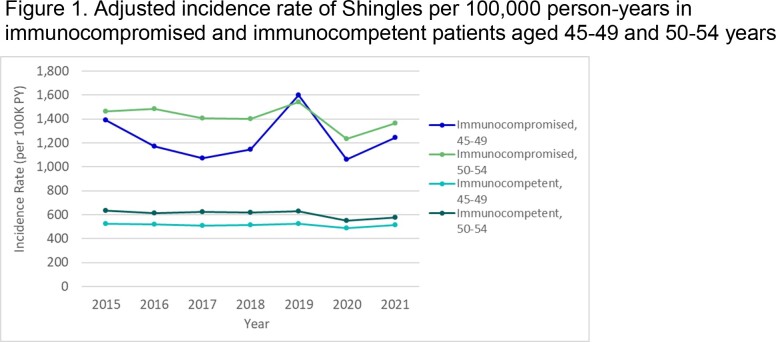

**Figure:**
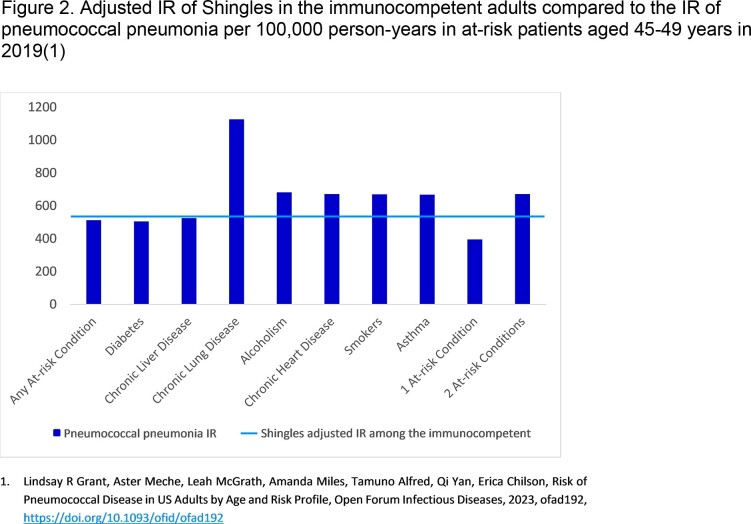

**Conclusion:**

Shingles incidence among adults aged 45–49 versus 50–54 was similar for IC patients. In recent years, IRs for immunocompetent patients aged 45–49 vs 50-54 were closer. There is an unmet need for shingles prevention in adults aged 45–49.

**Disclosures:**

**Amanda C. Miles, MPH**, Pfizer: Employee|Pfizer: Stocks/Bonds **Leah McGrath, PhD**, Pfizer: Employment|Pfizer: Stocks/Bonds **Verna Welch, PhD, MPH**, Pfizer Inc.: Stocks/Bonds

